# Development of the Shift Smartphone App to Support the Emotional Well-Being of Junior Physicians: Design of a Prototype and Results of Usability and Acceptability Testing

**DOI:** 10.2196/26370

**Published:** 2021-12-02

**Authors:** Isabelle Counson, Alexandra Bartholomew, Joanna Crawford, Katherine Petrie, Geetanjali Basarkod, Victoria Moynihan, Josie Pires, Rachel Cohen, Nicholas Glozier, Samuel Harvey, Samineh Sanatkar

**Affiliations:** 1 Black Dog Institute Randwick Australia; 2 School of Psychiatry Faculty of Medicine UNSW Sydney Kensington Australia; 3 Institute for Positive Psychology and Education Australian Catholic University North Sydney Australia; 4 Brain and Mind Centre Faculty of Medicine and Health University of Sydney Australia

**Keywords:** digital mental health, mHealth apps, help-seeking, junior physicians, co-design, user-centered design, mobile phone

## Abstract

**Background:**

Junior physicians report higher levels of psychological distress than senior doctors and report several barriers to seeking professional mental health support, including concerns about confidentiality and career progression. Mobile health (mHealth) apps may be utilized to help overcome these barriers to assist the emotional well-being of this population and encourage help-seeking.

**Objective:**

This study describes the development and pilot trial of the *Shift* mHealth app to provide an unobtrusive avenue for junior physicians to seek information about, and help for, well-being and mental health concerns, which is sensitive to workplace settings.

**Methods:**

A 4-phase iterative development process was undertaken to create the content and features of *Shift* involving junior physicians using the principles of user-centered design. These 4 phases were—needs assessment, on the basis of interviews with 12 junior physicians; prototype development with user experience feedback from 2 junior physicians; evaluation, consisting of a pilot trial with 22 junior physicians to assess the usability and acceptability of the initial prototype; and redesign, including user experience workshops with 51 junior physicians.

**Results:**

Qualitative results informed the content and design of *Shift* to ensure that the app was tailored to junior physicians’ needs. The *Shift* app prototype contained cognitive behavioral, mindfulness, value-based actions, and psychoeducational modules, as well as a tracking function that visualized patterns of daily variations in mood and health behaviors. Pilot-testing revealed possible issues with the organization of the app content, which were addressed through a thorough restructuring and redesign of *Shift* with the help of junior physicians across 3 user experience workshops.

**Conclusions:**

This study demonstrates the importance of ongoing end user involvement in the creation of a specialized mHealth app for a unique working population experiencing profession-specific stressors and barriers to help-seeking. The development and pilot trial of this novel *Shift* mHealth app are the first steps in addressing the mental health and support-seeking needs of junior physicians, although further research is required to validate its effectiveness and appropriateness on a larger scale.

## Introduction

Junior physicians exhibit levels of psychological distress and emotional exhaustion to a greater degree than their senior counterparts [1,2]. Junior physicians reported feeling impacted by a range of workplace-related stressors, including long working hours, a lack of breaks, and, at times, bullying and harassment [3,4]. A review of several prospective studies showed that individual factors, such as self-criticism, emotional instability, and a family history of psychopathology, are predictive of mental illness in junior physicians [5]. Furthermore, structural and personal barriers to help-seeking for mental health concerns have been noted, such as concerns about being reported to medical regulators and anxiety about *reversing roles* from being a physician to becoming a patient [6-8]. Australian data indicate that the most commonly reported barriers to professional help-seeking for depression among depressed physicians are privacy and confidentiality concerns [9].

Delays in receiving targeted treatment have the potential to compound and prolong symptoms of poor mental health as well as to increase the likelihood of developing comorbidities such as alcohol dependence [7,10]. Research suggesting a negative association between physicians’ psychopathology and best patient care practices highlights the implications of physicians’ mental health in the broader community [11-13]. Therefore, in addition to the adverse effects on the individual, it is in the wider public interest to support junior physicians in their transition into a demanding work environment and to help them seek and receive effective mental health care.

Although psychological interventions designed specifically to support physicians’ mental health are scarce, a recent meta-analysis found that interventions targeting physicians yielded small but significant reductions in symptoms of common mental disorder and suicidal ideation, particularly when therapeutic components were based on principles of cognitive behavioral therapy and mindfulness [14,15]. Of note, one study found that medical interns randomly assigned to a web-based cognitive behavioral therapy intervention group were 60% less likely to report suicidal ideation during their internship year than the comparator attention-control group [16]. Further research suggests that valued living and present-moment awareness components practiced in acceptance and commitment therapy and psychoeducational programs may be useful in reducing psychological distress in health care professionals and students [17-19].

Although the potential benefits of teaching these skills to junior physicians are clear, the practicality of such interventions can be challenging. Junior physicians are busy and regularly move between roles. New digital tools may be able to assist with these logistical challenges. Although mobile health (mHealth) apps have been shown to improve working populations’ mental well-being and willingness to seek help for mental health concerns [20-23], they have yet to be tested in well-controlled studies among physicians. Many physicians have already used mobile apps in the workplace to guide their management or prescription. A novel mHealth app using the same avenue as these professional development tools may be an acceptable way of delivering mental health support to junior physicians.

Previous research highlights the importance of a user-centered approach to developing mHealth app interventions [24-26] and, if designed for employees, additional factors, such as the workplace environment, should be taken into account [27,28]. As such, mHealth apps involving workplace considerations pose additional constraints on the app development process to ensure that content is effective, adequately delivered, and suits the target working population. User-centered design approaches involve iterative phases of prototyping, ideally employing co-design and end user feedback, and involve consideration of the user at every stage of the design process to ensure that the intervention meets their needs [29,30]. This, in turn, has been reported to maximize users’ engagement with, and adherence to, an mHealth intervention, and hence its impact [31].

This paper describes the process of developing *Shift*, a self-guided mental health and help-seeking smartphone app for junior physicians located in New South Wales, Australia. To our knowledge, this is the first mHealth app designed specifically to support the mental health of junior physicians. We used a user-driven and iterative development process, employing the principles of user-centered design and a multiphase process. This paper presents the 4-phase *Shift* app development process and how this process incorporates knowledge and feedback derived from qualitative assessments, pilot-testing, stakeholder and expert consultations, and user experience workshops with the target population.

## Methods

### Overview

There were 4 project phases as follows: phase 1, needs assessment through qualitative end user interviews; phase 2, *Shift* app prototype development; phase 3, pilot-testing of the *Shift* app prototype; and phase 4, generation of an updated version of the *Shift* app. In preparation for phase 1, consultations with a range of stakeholders were conducted (including junior physician managers, providers of support services for junior physicians, and professional organizations related to junior physicians in New South Wales) to examine the existing mental health support services for junior physicians, and facilitators of and barriers to engagement with these services. This was to ensure that the app development complemented existing support and provided up-to-date information on available services. Furthermore, our broader research team had previously developed *HeadGear,* an mHealth app that has been found to be effective in male-dominated working populations [21,32,33]. The intervention component in *HeadGear* was delivered in a *30-day challenge* format, which successively unlocked psychoeducational material, as well as behavioral activation, goal-setting, and mindfulness techniques. Hence, one option available to our team was to utilize the *30-day challenge* format of the evidence-based *HeadGear* app and to modify the clinical content to meet the needs of junior physicians. To this end, the interviews with junior physicians in phase 1 included a question about their attitudes toward a *30-day challenge* in an app to support the well-being of junior physicians.

### Phase 1: Needs Assessment Through Qualitative Interviews

The objective of the qualitative component of this project was to inform the design and development of clinical content and to ensure that the app is tailored to the specific needs, characteristics, and challenges faced by this unique user group. Specifically, the interviews aimed to (1) identify the main stressors and challenges for junior physicians, both at and outside of work; (2) explore their attitudes toward a mental health app; (3) identify facilitators of and barriers to their use of, interest, and engagement in a mental health app; (4) assess current use of general apps; and, finally, (5) to identify their suggestions, preferences, and dislikes or unwanted features that they felt would support junior physicians’ mental health and well-being. The qualitative component was granted full ethical approval by the South Eastern Sydney Local Health District Human Research Ethics Committee (protocol #: 18/140, HREC/18/POWH/321). A qualitative analysis of the interviews focusing on the experiences of junior physicians is reported in detail elsewhere [34]. This paper presents specific app-related items and findings from interviews.

Participants were recruited from 2 metropolitan hospital sites in the Sydney area between July and September 2018. Advertising took place via email invitations distributed by medical supervisors, through email and social media announcements to professional organizations related to junior physicians, and through on-site hospital visits by members of the research team (JC, KP, and GB); 41 junior physicians expressed interest in the study, of whom 12 provided written informed consent and were recruited for one-on-one face-to-face interviews (conducted by JC). Participants included 9 women and 3 men aged between 24 and 35 years. The sample was evenly spread across the early stages of training and comprised interns (n=3), residents (n=5), and registrars (n=4). The majority had studied medicine in Australia and were generally 3-5 years into their clinical training.

Data on app-related items were analyzed using a thematic analysis approach [35] informed by grounded theory and Massey *emergent* approach [36]. In an iterative process undertaken by one researcher (KP), all transcripts were reviewed closely to generate an initial first-level coding framework. Through subsequent refinements through discussion with co-authors, broader second-level themes related to the app issues were identified, and subthemes and common suggestions were listed under each of the 4 main issues of interest, summarized below in the Results section.

### Phase 2: Prototype Development

On the basis of the recommendations made by junior physicians in the qualitative interviews and consultations with stakeholders in line with evidence from recent literature [14,17,18], new clinical content for the *Shift* app was written by 1 psychologist, 2 clinical psychologists (JC and RC), 2 psychiatrists (SH and NG), and 1 researcher (GB).

### Phase 3: Pilot-Testing

Pilot-testing was conducted in October and November 2019 to examine the usability and acceptability of the *prototype version of the Shift app*. Trends in depression and anxiety symptom severity, as well as changes in help-seeking intentions before and after using the *Shift* app prototype over a 4-week period were also examined. This study was approved by the South Eastern Sydney Local Health District Human Research Ethics Committee (protocol # 2019/ETH00318).

**Figure 1:**
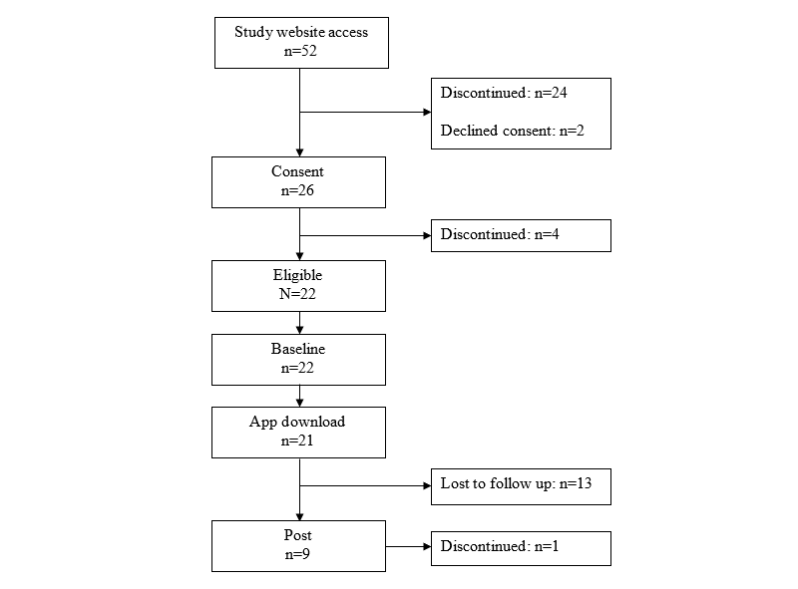
Flow chart describing how participants progressed through the pilot study phases.

Two New South Wales hospital sites, one regional and one metropolitan, issued recruitment calls via email messages on behalf of the research team to junior physicians at the intern, resident, or registrar levels. The eligibility criteria were current employment as junior medical officers in New South Wales and ownership of an internet-enabled smartphone with an Apple or Android operating system. A total of 52 candidates accessed the study website, of which 50% (26/52) consented to participate. A final sample of 22 participants (13/22, 59% women; mean age 29, SD 4.1 years) entered the pilot study and completed a baseline assessment. A diagram of the participant flow is shown in Figure 1.

A baseline questionnaire assessed basic demographic information (age, gender, level of training, and regional or metropolitan placement) and symptoms of depression (Patient Health Questionnaire 9-item) and anxiety (General Anxiety Disorder scale 7-item) over the past 2 weeks. Participants indicated their depression and anxiety symptoms on a 4-point Likert-type response scale ranging from 0 (*not at all*) to 3 (*nearly every day*). In addition, participants indicated their previous and recent help-seeking intentions for mental health problems (eg, “If you were to develop a mental health issue, how likely would you be to seek help from a GP or mental health professional [eg, psychologist/psychiatrist]?”) on an 11-point Likert-type response scale ranging from 0 (*not at all likely*) to 10 (*very likely*). After completion of the baseline questionnaire, participants were given instructions on how to download the *Shift* app onto their mobile phones. The app automatically recorded usage metrics, such as the number of log-ins and challenges or sessions completed.

After 1 month, participants were invited via email to complete a poststudy questionnaire reassessing their current depression and anxiety symptom levels and help-seeking intentions. At the poststudy assessment point, participants were further asked to respond to a battery of purpose-built questions relating to the usability and acceptability of the *Shift* app prototype (eg, “Was the app interesting/engaging?”) on a 5-point Likert-type scale ranging from 1 (*not at all*) to 5 (*completely*) and to the overall satisfaction rating of the app on a 5-point Likert-type scale ranging from 1 (*low*) to 5 (*high*). A modified version of the System Usability Scale was also administered to obtain an objective indication of the overall ease of use of the app.

### Phase 4: Shift App Redevelopment

It was anticipated that a final phase of design would be required after the initial pilot-testing of the *Shift* app. A series of 3 user-consultation workshops (N=51) at 2 metropolitan hospitals, driven by a lead user experience designer (VC), helped develop any final changes and responses to insights gained from the pilot-testing. The feedback of junior physicians participating in the workshops was collected using an interactive prototype containing screen mock-ups and questions aimed at testing potential changes and solutions.

## Results

### Phase 1: Needs Assessment Through Qualitative Interviews

#### Attitudes Toward a Mental Health App for Junior Physicians

All participants reported that they owned a smartphone and used apps multiple times each day. Half of the sample had already tried at least one mindfulness app, and 2 had previously used a web-based mental health app. Although the use of work-related apps was common, the use of such apps was centered on communication and medical information–seeking and reference material. A total of 83% (10/12) participants reported to be *very or somewhat interested* in the idea of an app to support the mental health of junior physicians and provided positive endorsement for the idea in principle and for its potential to benefit the population. Most of the participants endorsed use of the app for both prevention of and early intervention in mental illness and suggested the inclusion of directions to support and treatment services for those with more severe mental illness. Although no participants were directly opposed to the idea of an mHealth app to support junior physicians’ mental health, 2 participants questioned whether fellow junior physicians would use an mHealth app or to what extent they would benefit from it, particularly if physicians were not currently experiencing symptoms of mental illness (“no one takes their medicine when they are feeling good”).

#### App Naming, Content, and Feature Suggestions

*Shift* was named based on participants’ views that the name of the app should be unobtrusive and not obviously related to mental health. This term refers to both shift work, which is one of the most common stressors in this population, and to shifting cognitions and behaviors to promote better mental health and well-being, in line with cognitive behavioral and mindfulness principles.

Participants made numerous suggestions for clinical app contents, including mindfulness, sleep strategies, mood monitoring, behavioral activation, and cognitive therapy targeting specific situations commonly faced by junior physicians. The provision of strategies to deal with problematic work situations was often seen as having a stress-buffering and destigmatizing effect. A list of recommendations for the broad content areas provided by participants is presented in Table 1.

**Table 1:** Qualitative interview participants’ overview of main content recommendations for *Shift* and whether recommendations were adopted in successive versions of the app (N=12).

Recommended content components	Version 1	Version 2
Cognitive behavioral therapy	✓	✓^a^
Sleep hygiene	✓	✓
Mindfulness and stress management	✓	✓
Goal-setting	✓	✓
Pleasant activity scheduling	✓	✓
Practical lifestyle strategies	✓	✓^a^
Problematic work situations	✓	✓^a^
Hand over tips for changing terms or hospitals		
Stories from junior physicians		✓^a^

^a^Indicates an addition or improvement compared with the previous version.

All participants provided positive feedback about the idea of a *30-day challenge*, with some reporting that they were more likely to use this feature as it was time-limited and seemed achievable with a set end point and small regular goal-oriented challenges. Several participants suggested graphical feedback on symptom trackers that compared multiple outcomes over time to *appeal to the scientifically minded*. A list of desired mHealth app features, as expressed by the participant sample, is presented in Table 2.

**Table 2:** Qualitative interview participants’ desired features for *Shift* and whether recommendations were adopted (N=12).

Desired features	Version 1	Version 2
Logical, clear app structure with clear user flow	✓	✓^a^
Simple layout, easy to navigate quickly	✓	✓^a^
Default private option—no linkage to social media	✓	✓
Quick start option, easy access log in	✓	✓^a^
Skip function; ability to return to modules later	✓	✓^a^
Provision of both text and audio formats	✓	✓
Symptom tracker function with graphics showing charts	✓	✓^a^
At log in, quick tick box of symptom self-assessment	✓	✓^a^
Centralized access to many things from one place	✓	✓^a^
Notifications and reminders should be optional	✓	✓

^a^Indicates improvement compared with the previous version.

#### Facilitators of and Barriers to App Use

The main app use barrier reported by the participants was that the app would feel like another chore when participants were already time-poor and working long hours. Although most participants expressed concerns about confidentiality, deidentification, and minimal information-sharing, 92% (11/12) of participants reported that they would still provide their name and email address to register to a mental health app.

The main facilitators of app engagement that the participants reported were the app being quick and easy to use and having added-value features that would distinguish the app over others (*that’s targeted to medics, that other apps aren’t going to address*). Most participants reported that they would be happy to use the app quite frequently, such as every morning or every few days, but only if the sessions were very brief.

### Phase 2: Prototype Development

The *Shift* app was developed for use in Android and Apple operating systems. The main features of the app are the following: (1) therapeutic and psychoeducational modules, (2) provision of contact details to mental health organizations and workplace resources, (3) mood and habit tracking, and (4) brief symptom assessments. *Shift* delivers content through a variety of text, audio, video, and graphical displays.

On the basis of previous research on the therapeutic benefits of cognitive behavioral, mindfulness, and value-based action components for medical professionals [14,15] and guided by preferences expressed in phase 1 qualitative interviews, the *Shift* app was developed employing cognitive behavioral principles of thought evaluation (ie, identification, evaluation, and modification of unhelpful thoughts) and engagement in valued action (ie, values-consistent patterns of action) adopted from acceptance and commitment therapy. Relaxation techniques (eg, progressive muscle relaxation) based on mindfulness and stress management practices were also incorporated to lessen the impact of stressful life events or daily stressors. Cognitive behavioral, value-based, and mindfulness modules were generated and presented in a 30-day challenge format. Each challenge was designed to take approximately 3-4 minutes to complete.

In addition, a suite of psychoeducational modules (*sessions*) was developed, including informational content on common mental health disorders, avenues through which to seek help for mental health concerns, and suggestions on how to incorporate relevant strategies, such as how to adjust to shift work. Psychoeducational content included mental health, help-seeking, and workplace information, such as depression, anxiety, mandatory reporting, at home and workplace avenues for seeking help, workplace bullying, adjusting to rural and regional placements, exams and interviews, and sleep health.

A tracking tool and symptom screening options were designed to allow users to capture daily snapshots of how they were faring and, in the case of the tracking function, build a visual tool to observe variations in mood and behavioral patterns over time.

The clinical content and design drafts of the app prototype were modified based on user experience feedback. User experience experts and psychologists worked with 2 junior physicians to refine the user pathways, functionality, color palette, and design and to modify the clinical content (eg, examples of scenarios used in cognitive therapy). Tables 3 and 4 outline the contents of the resulting *Shift* app prototype, which were organized into challenges and sessions.

**Table 3:** Organization of challenge topics in the *Shift* app prototype version.

Therapeutic type and challenge day or days			Topic name		Type
**Mindfulness**					
	2	Introduction to mindfulness		Video	
	3	Seeing the horizon		Audios	
	9	Grounding anchor		Audios	
	16	Calming breath		Audios	
	20	Loving-kindness		Audios	
	24	Cargo thoughts		Audios	
	26	Breathing wind		Audios	
	28	Lapping ocean		Audios	
**Value-based**					
	4	Introduction to values and values as a physician		Video	
	5	Strive for five		Text	
	6, 12, 17, 21, 27	Scheduling meaningful actions		Text	
**Cognitive behavioral**					
	7	Introduction to unhelpful thoughts		Video	
	8	Unhelpful thoughts		Text	
	11	Cognitive biases		Text	
	14	Introduction to thought challenging		Video	
	15	Thought challenging		Text	
	22	Worry decision tree		Text	
	23	Cognitive therapy review		Video	
**Positive psychology**					
	10	Gratitude		Text	
	13	Getting active		Text	
	18	Social support		Text	
	19	Help a friend		Text	
	25	100 enjoyable activities		Text	
	29	Planning for the future		Text	
**General**					
	1	Checkup		Text	
	30	Putting it all together		Video	

**Table 4:** Organization of session topics in the *Shift* app prototype version.

Topic			Type
**Sleep and fatigue**			
	Sleep health	Text	
	Adjusting to shift work	Text	
**Common mental health problems**			
	Depression	Text	
	Anxiety	Text	
	Burnout	Text	
	Posttraumatic stress	Text	
	Alcohol and other drugs	Text	
**Getting help**			
	Get help now	Text	
	Dealing with intense emotions	Text	
	How to seek help: workplace avenues	Text	
	How to seek help: nonworkplace avenues	Text	
	Mandatory reporting	Text	
**Common issues for JMOs^a^**			
	Exams and interviews	Text	
	Work-life balance	Text	
	Adjusting to rural and regional placements	Text	
	Bullying in the workplace	Text	
	Dealing with the death of a patient	Text	
	Calling for a consult	Text	
	Feeling inadequate	Text	

**^a^**JMO: junior medical officer.

### Phase 3: Pilot-Testing

#### App Acceptability and Usability

As shown in Table 5, the median responses to questions relating to overall app rating, content understandability, appropriateness, and usefulness were all on or above the midrange of the response scales. The overall system usability rating was 84.72 (SD 8.33), which was above the average score of 70 across technological tools more generally [37] and comparable with an average score of 77 reported for common smartphone apps and tablets [38]. Tables 6 and 7 present a breakdown of participants’ usefulness ratings of *Shift* challenge and session components. Participants rated the mindfulness challenges favorably and rated the general and value-based components least favorably. Among the session topics, the sleep and fatigue information components received the highest usefulness ratings, while the other sessions were rated lower or were not attempted.

**Table 5:** Pilot trial participants’ responses to the usability and acceptability of the *Shift* app prototype (N=9)^a^.

Item	Values, median (range)	Values, minimum-maximum
How well did you understand the content of the app?	5 (2)	3-5
Was the app content appropriate for you?	5 (3)	2-5
Was the app interesting/engaging?	4 (2)	3-5
Do you feel that the app has helped you improve your mental health?	3 (2)	1-3
Would you recommend this app to other junior physicians?	4 (2)	3-5
What is your overall rating of the app?	3 (3)	2-5

^a^Response scales ranged from 1 to 5.

**Table 6:** Pilot trial participants’ usefulness ratings of the *Shift* app prototype 30-day challenge contents (N=8)^a^.

Challenge type and topic name^b^		Values, median (range)	Values, minimum-maximum
Mindfulness		4 (5)	0-5
Value-based		0 (5)	0-5
Cognitive behavioral		3 (5)	0-5
**Positive psychology**			
	Gratitude	3 (5)	0-5
	Getting active	1.5 (5)	0-5
	Social support	2.5 (5)	0-5
	Help a friend	0 (4)	0-4
	Enjoyable activities	3 (5)	0-5
	Planning for the future	1.5 (5)	0-5
General		0 (4)	0-4

^a^Response scales ranged from 0 to 5, where 0 indicates unattempted components, 1 indicates low perceived usefulness, and 5 indicates high perceived usefulness.

^b^Only positive psychology challenge topics were assessed individually because of the distinctiveness of each topic in this category.

**Table 7:** Pilot trial participants’ usefulness ratings of the *Shift* app prototype session contents (N=8)^a^.

Session type and topic name			Values, median (range)		Values, minimum-maximum
**Sleep and fatigue**					
	Sleep health	3 (5)		0-5	
	Adjusting to shift work	3 (5)		0-5	
**Common mental health problems**					
	Depression	1.5 (4)		0-4	
	Anxiety	2.5 (5)		0-5	
	Burnout	1.5 (5)		0-5	
	Posttraumatic stress	0 (4)		0-4	
	Alcohol and other drugs	0 (4)		0-4	
**Getting help**					
	Intense emotions	0 (4)		0-4	
	Workplace avenues	1 (5)		0-5	
	Nonworkplace avenues	1.5 (5)		0-5	
	Mandatory reporting	0 (5)		0-5	
**Common issues for JMOs^b^**					
	Exams and interviews	0 (3)		0-3	
	Work-life balance	1.5 (5)		0-5	
	Remote placements	0 (4)		0-4	
	Workplace bullying	0 (5)		0-5	
	Death of a patient	0 (5)		0-5	

^a^Response scales ranged from 0 to 5, where 0 indicates unattempted components, 1 indicates low perceived usefulness, and 5 indicates high perceived usefulness.

**^b^**JMO: junior medical officer.

#### App Use

Of the 22 participants, 95% (21) downloaded *Shift* and used the app at least once. The mean number of log-ins was 5.24 (SD 5.93) and participants spent an average of 28 minutes (SD 52.7) on the app, although the large SD indicated that there was considerable variability in use times. The median use time was 11 minutes (range 199.35). Participants completed an average of 5 challenges (SD 7.75) and spent 3.24 minutes working through these challenges (SD 4.53). Sessions were used less frequently (mean 1.81, SD 2.56), and less time was spent on sessions (mean 1.38, SD 1.46 minutes).

#### Symptoms Change

Wilcoxon signed-rank tests indicated that depression (*Z*=−1.38; *P*=.168) and anxiety (*Z*=−1.05; *P*=.293) scores slightly decreased, albeit not significantly, and that help-seeking intentions were largely unchanged (*Z*=−.38; *P*=.705) over the 1-month period of app use.

### Phase 4: Shift App Redevelopment

On the basis of the results of the pilot, a major redesign of the prototype was conducted to create a more user-friendly and user-driven learning experience. The pilot study results indicated that the first version of the app did not engage junior physicians sufficiently well, which may have been an important factor contributing to small effect sizes in symptom change and help-seeking scores. A series of 3 user-consultation workshops (N=51) at 2 metropolitan hospitals helped finalize the proposed changes to the prototype. These were (1) layout and design improvements, (2) increased personalization and ease of access, (3) updates of clinical contents, (4) the inclusion of self-reflection activities, and (5) the adoption of more meaningful and relatable wording [6].

A streamlined login process with the inclusion of a biometric security system (ie, fingerprint or face ID authentication) was incorporated to facilitate use after app download. Personalization enhancements were achieved through significant changes in the presentation of the app. Importantly, the 30-day challenge structure was removed, and challenges and sessions were organized under a general overview of the topics. In this view, users were directed to contents through headings named *Mental Health*, *Getting Help*, *Lifestyle*, and *Work*. The *challenge* concept was maintained by incorporating an option for users to set their own weekly targets (ie, number of activities to complete each week) during the app on boarding process. With this new functionality, users could choose achievable goals relating to their app use while still encouraging regular use of the app.

**Figure 2:**
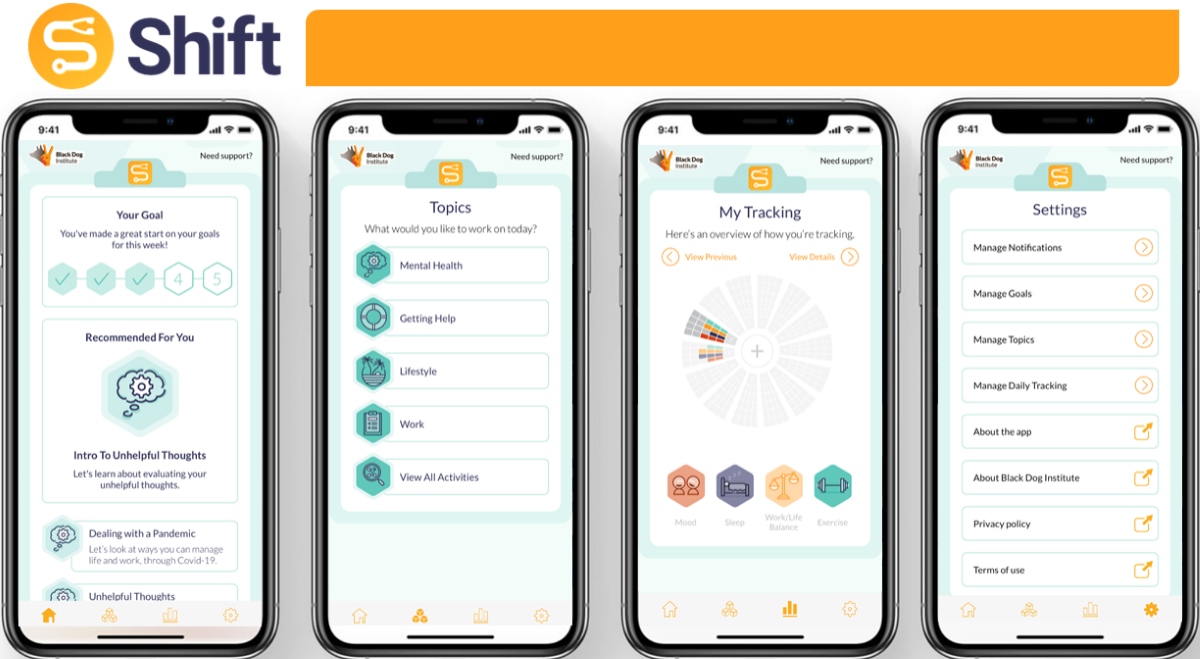
Visual examples of the most recent *Shift* version Home, Topics, Tracking, and Settings screens.

The previously limited line-graph tracking function was completely redesigned to become more interactive and visual, as well as allowing a new option of *work/life balance*. To accommodate these changes, thorough layout and presentation updates were made, as well as enhancing the interactivity with a complimentary day-by-day view to show which activities were completed on which dates. Consistent with the user experience workshop feedback, new modules on exercise and diet were incorporated into the app. Two additional modules were developed in response to the COVID-19 pandemic. Informational sessions were extended with the inclusion of example stories from junior physicians and adjunct brief empirical evaluations (*symptom screeners*, for example, the Patient Health Questionnaire–2-item, General Anxiety Disorder scale–2-item). Example stories, based on recommendations put forward by junior physicians in qualitative interviews (Table 1), illustrated mental health challenges and invited users to elaborate on symptoms. The mHealth app contents were finally proofread by 2 clinical psychologists, 3 researchers (IC, AB, and SS), and 1 digital learning designer (JP) to ensure the provision of up-to-date clinical and psychoeducational contents in a way that facilitates learning [39,40]. The structure and design graphical guides of the novel *Shift* version are provided in Figure 2.

## Discussion

### Principal Findings

This paper describes the development of an mHealth app, *Shift*, designed to support the mental health and help-seeking of junior physicians. In line with gold standard recommendations on the importance of user-centered design principles [26], the 4-phased app development process (ie, semistructured interviews, prototype development, pilot-testing, and app redevelopment) focused on a participatory approach to promote effective engagement and facilitate cognitive, affective, and behavior changes of junior physicians. Junior physicians were involved at every stage of this process through qualitative interviews, user experience workshops, and participation in pilot-testing. The aim of this approach was to create, deliver, and refine content in a way that was acceptable, effective, and engaging to end users.

Pilot-testing revealed several issues with the delivery of in-app content components to junior physicians. Although a successive, day-by-day delivery of therapeutic content has been successfully employed in a previous working population sample [21] and was generally viewed favorably by junior physicians in qualitative assessments, preliminary use data indicated that this format failed to engage junior physicians in practice. Inspection of app use data revealed that discontinuation of the 30-day challenge tended to appear around day 5, which was a generally lower rated, value-based activity. With the 30-day challenge format, users were unable to skip challenge topics or change the order of challenges, possibly facilitating the discontinuation of app use. As a time-poor, well-educated group, junior physicians may be more insistent on being able to choose their own modules from other working populations. In addition, informational sessions were underutilized in comparison to challenge content, possibly due to their less-prominent positioning within the app. Feedback on challenge and session components indicated considerable variability in the favorability ratings of the contents. This observation highlights 2 key aspects. First, even when app components are generated based on qualitative data from focus groups, their use in the real world needs to be tested. Second, simply modifying the modes of content delivery (ie, 30-day challenge structure) from one evidence-based app to suit another working population is not always successful. To meet the needs of a population of junior physicians, a new app structure needs to be developed. Therefore, in phase 4, the app was adjusted with the help of junior physicians across successive user experience workshops to enhance the overall experience and encourage engagement. The main changes included streamlining login and onboarding procedures and categorizing contents by topics, which allowed for the personal selection of modules and for an updated design and learning experience.

### Strengths and Limitations

The 4-phase process emphasizes the need for customization for end users. In line with previous research, this project illustrates the role of usability testing in the development of a digital intervention tool [24,41]. Using participatory mixed methods, such as qualitative and quantitative assessments, to involve end users at all stages of the product development process was fundamental in our attempt to create a digital solution that allowed for the pursuit of multiple outcomes, such as cognitive behavioral, psychoeducational, or providing contact details to relevant specialized services [26]. Furthermore, our development process suggests that user experience and learning designers are critical in translating methods proven useful in face-to-face settings in the digital arena. The establishment of a multidisciplinary team including academics, clinicians, and digital experts helped incorporate the suggestions and feedback put forward by junior physicians into an mHealth app intervention environment.

This study had several important limitations. First, the *Shift* mHealth app has not been evaluated for its effectiveness to date. Future studies are required to establish whether it is indeed a useful tool to reduce or prevent the onset of common mental health symptoms in junior physicians. Similarly, it needs to be established whether help-seeking intentions or actions improve after using *Shift*. Second, although the increased personalization of the novel *Shift* version is expected to increase engagement, the freedom to choose modules may unintentionally facilitate avoidance behaviors or choice overload and thus potentially minimize exposure to beneficial content. Thus, use behaviors and outcomes should be thoroughly inspected to predict which use patterns constitute effective engagement if such a pathway indeed exists. Finally, *Shift* focuses on individual-level change factors. However, to address distress among junior physicians in a comprehensive manner, structural change programs including organization-level solutions are likely to be required alongside the delivery of interventions directed at an individual, such as the app [14].

### Conclusions

The integration of new technology in the creation of workplace and personal well-being programs constitutes an easily accessible and cost-effective approach to addressing mental health concerns. Digital mental health programs provide a potential solution to engaging hard-to-reach, time-poor, and potentially help-seeking averse junior physicians in a way that does not exasperate confidentiality concerns around discussing mental health problems face-to-face. To our knowledge, *Shift* is the first initiative of its kind in that it aims to deliver mental health and support resources to junior physicians through unobtrusive mHealth app technology. This study describes an innovative, multiphase, and multidisciplinary user-driven design process that was undertaken to ensure a match between the app and the needs and barriers faced by junior physicians. Further research is planned to examine whether *Shift* proves useful for a substantial number of junior physicians.

## Abbreviations

mHealth: mobile health
